# Supplementary material for: Insights into the Underlying Mechanism of Ochratoxin A Production in Aspergillus niger CBS 513.88 Using Different Carbon Sources
Source: Toxins (Basel). 2022 Aug 12;14(8):551. doi: 10.3390/toxins14080551 (PMC9415321; doi:10.3390/toxins14080551)
Supplement: Supplementary file 1 [file toxins-14-00551-s001.zip › toxins-1837911-supplementary.pdf]

Table S1. The significantly changed TFs that overlapped in the three OTA-inducing conditions (YEA, YES, YEG) compared to non-inducing condition (YEP).

Gene_ID	Family	Description	SGD	Description
**Up-regulated**				
An15g00120	C2H2	C2H2 transcription factor	*azf1*	Regulates expression of genes involved in carbon metabolism, energy production, cell wall organization and cell cycle
An08g10980	ZnCys	hypothetical protein ANI_1_2502074	*asg1*	Regulator involved in the stress response, utilization of fatty acids and accumulation of lipids
An01g13080	ZnCys	C6 transcription factor	*asg1*	Regulator involved in the stress response, utilization of fatty acids and accumulation of lipids
An04g04490	ZnCys	hypothetical protein ANI_1_1838184	*put3*	Regulates proline utilization genes
An15g05100	ZnCys	C6 transcription factor	*ecm22*	Regulates transcription of sterol biosynthetic genes
An15g05310	ZnCys	C6 zinc finger domain protein	*upc2*	Regulates transcription of sterol biosynthetic genes
An18g00320	ZnCys	Zn(II)2Cys6 transcription factor	*upc2*	Regulates transcription of sterol biosynthetic genes
An04g08080	ZnCys	C6 transcription factor	*rgt1*	Regulates expression of several *HXT* genes in response to glucose
An12g06190	ZnCys	Zn(II)2Cys6 transcription factor	*stb4*	Regulates expression of genes encoding transporters
An18g00850	NEG	hypothetical protein ANI_1_1016164	YKL070W	unknown function
An08g11000	ZnCys	hypothetical protein An08g11000	/	/
An15g05080	ZnCys	hypothetical protein ANI_1_1562134	/	/
An13g03030	ZnCys	hypothetical protein ANI_1_762114	/	/
An01g11310	ZnCys	hypothetical protein ANI_1_3052014	/	/
An11g00290	ZnCys	hypothetical protein ANI_1_1536094	/	/
An13g01420	ZnCys	C6 transcription factor	/	/
An14g05790	ZnCys	C6 transcription factor	/	/
An04g09790	ZnCys	fungal specific transcription factor	/	/
An09g01870	ZnCys	fungal specific transcription factor	/	/

		domain protein		
An08g03770	ZnCys	C6 zinc finger domain protein	/	/
An17g00650	ZnCys	Zn(II)2Cys6 transcription factor	/	/
An07g08880	bZIP	bZIP transcription factor	/	/
An13g03530	HOMEOLIKE	transposase	/	/
An12g07000	HOMEOLIKE	transposase	/	/
An12g04610	OBFOLD	endoglucanase-4	/	/
An18g00850	NEG	hypothetical protein ANI_1_1016164	/	/
**Down-regulated**				
An12g10230	bZIP	hypothetical protein ANI_1_2318104	*aca1*	Important for carbon source utilization
An09g02620	ZnCys	unnamed protein product	*gal4*	Activating GAL genes
An12g00840	ZnCys	unnamed protein product	*gal4*	Activating GAL genes
An01g10540	C2H2	regulatory protein BrlA	*msn4*	Regulator involved in the stress response
An01g02050	ZnCys	unnamed protein product	*asg1*	Regulator involved in the stress response; regulates utilization of fatty acids and accumulation of lipids
An11g02150	ZnCys	unnamed protein product	*aro80*	Activates transcription of aromatic amino acid catabolic genes
An03g06510	ZnCys	unnamed protein product	*arg81*	Regulates transcription of arginine-responsive genes
An14g07040	ZnCys	Zn(II)2Cys6 transcription factor	*upc2*	Regulates transcription of sterol biosynthetic genes
An04g06940	ZnCys	Transcriptional activator of proteases PrtT	*upc2*	Putative Zn(II)2Cys6 motif containing transcription factor
An04g07400	ZnCys	Putative C6 zinc finger transcription factor	*ume6*	Negatively regulates of early meiotic genes
An02g01080	ZnCys	unnamed protein product	/	/
An03g06370	ZnCys	hypothetical protein ANI_1_1544034	/	/
An15g07070	Lambda	cyanate hydratase	/	/
An14g05840	WING	unnamed protein product	/	/

Table S2. All primers used in this study.

Primer name	Primer sequence (5′-3′)
An08g06800-F	GATCAAGACCGCAGCCATCTCC
An08g06800-R	TCGCCTTCAGAATGTCCTCAATGC
An14g06010-F	CTGAAGGTGTTGGAGCGGTTGG
An14g06010-R	CCTTGGTGAGCGGGATGACATATTC
An15g02460-F	TTCTGTGATCGTGGAGACACTGTTG
An15g02460-R	ACATCAGGTCGCAAGCCATTCG
qgpdA-F	GGCTTCGGTCGTATCGGA
qgpdA-R	GTAGGCAGCATAGTGGGTC
pks-F	GCTTCCAATCAGTCCGTCCAGTC
pks-R	TCCTCTCCATCTGTATCCGCTGTAG
p450-F	AGAGTTGGTGAGTCGGGCTTCC
p450-R	TGTCATAGTATGGGCGGTAGTCTCC
nrps-F	AGGCTGTATGCTCGTGGGAT
nrps-R	GCCACAGCGTTGCAGTATCT
hal-F	CCGGTATCTGCCAGCTGGAA
hal-R	GTACTTGGTGCTGGCGATGC
bzip-F	GGGTATCTTATCGTCGGTTGAGCTG
bzip-R	GTGATCTGGATGCCCTCGTGATTC
An08g08920-F	TTGTTCCAGGCGTTGCTCCATC
An08g08920-R	CGCTGGTAAGGACAGTACACTTGG
An09g00820-F	GTTCCTCGGCGATCACGGTTAC
An09g00820-R	CCATGTTATTCCCATCCCAGGTCTG
An14g00690-F	CGAAGACGGTTGCGAAGAATGTTG
An14g00690-R	ACTCGGCGGGTAGCAATTTGTG
An02g07930-F	TCAGTCGCAAATGTTCACCCAGATC
An02g07930-R	GTCATTAGCCTCCGCAGTGTTCTC
An08g09150-F	AGTGTCTTGCCTGGGCGAATTAC
An08g09150-R	AACCTCCTTCCGACCCGTCATC
An16g00630-F	CTTCTTCACCGCCTCCCAACTTG
An16g00630-R	CTTCACCGAATACGCCCTGGAATAG

Table S3. Sequences of *Gal4* binding sites on the predicted promoter.

OTA biosynthetic genes	Sequence		
An15g07920(*pks*)	AGG	CAGCATTCCCA	CCG
	CGG	AGTGTTATGAG	CCG
An15g07900(*p450*)	AGG	GCCTTGCGTCG	CCG
	AGG	CATTGGAACTA	CCG
	CGG	CCCGCAGCCAA	CCG
An15g07910(*nrps*)	AGG	ACCCCTGGTTCA	CCG
	CGG	GCGATGTCATCA	CCG
	CGG	GTACACCAGCTA	CCG
An15g07880(*hal*)	CGG	TTGGCTGCGGG	CCG
An15g07890(*bzip*)	AGG	GTAGAGAAGG	CCG
	AGG	CGCGTGCAAG	CCG
	CGG	AGCGGAGGAAGC	CCG
	CGG	CCGCGATACA	CCG
	CGG	TAGCTGGTGTAC	CCG
	CGG	TGATGACATCGC	CCG
	CGG	CTTTCTTTTA	CCG

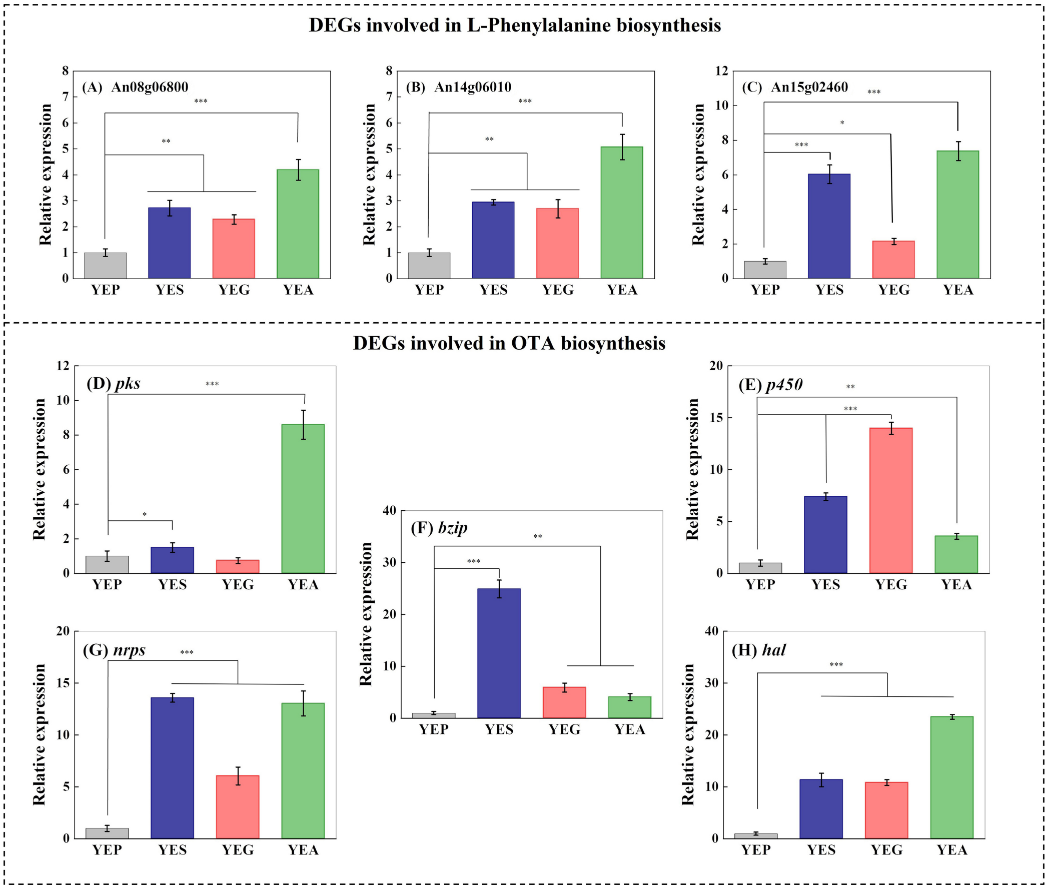

Figure S1. qRT-PCR analyses of differentially expressed genes in OTA precursor phenylalanine and OTA biosynthesis in the presence of three OTA-inducing carbon sources. $*t < 0.05$, $**t < 0.01$, $***t < 0.001$.
